# Cervical Vestibular-Evoked Myogenic Potentials: Norms and Protocols

**DOI:** 10.1155/2012/913515

**Published:** 2012-04-08

**Authors:** Suwicha Isaradisaikul, Niramon Navacharoen, Charuk Hanprasertpong, Jaran Kangsanarak

**Affiliations:** Department of Otolaryngology, Faculty of Medicine, Chiang Mai University, 110 Intawaroros Road, Sriphum, Mueang, Chiang Mai 50200, Thailand

## Abstract

Vestibular-evoked myogenic potential (VEMP) testing is a vestibular function test used for evaluating saccular and inferior vestibular nerve function. Parameters of VEMP testing include VEMP threshold, latencies of p1 and n1, and p1-n1 interamplitude. Less commonly used parameters were p1-n1 interlatency, interaural difference of p1 and n1 latency, and interaural amplitude difference (IAD) ratio. This paper recommends using air-conducted 500 Hz tone burst auditory stimulation presented monoaurally via an inserted ear phone while the subject is turning his head to the contralateral side in the sitting position and recording the responses from the ipsilateral sternocleidomastoid muscle. Normative values of VEMP responses in 50 normal audiovestibular volunteers were presented. VEMP testing protocols and normative values in other literature were reviewed and compared. The study is beneficial to clinicians as a reference guide to set up VEMP testing and interpretation of the VEMP responses.

## 1. Introduction

Vestibular-dependent myogenic responses to intense sound were first described by Bickford et al., in 1964 [1]. In 1994, Colebatch, Halmagyi, and Skuse established a reliable procedure to record myogenic potentials from the sternocleidomastoid (SCM) muscle evoked by clicks. A biphasic positive negativity (p1-n1) occurred in normal subjects but was abolished in patients who underwent selective vestibular nerve section [2]. In 1995, Halmagyi and Colebatch reported the responses that were not of lateral canal origin and the term “Vestibular-evoked myogenic potentials” (VEMP) has been widely used since then [3].

VEMP assesses vestibular function through the vestibulocollic reflex (VCR). The VCR arc includes the receptor (the saccule), the afferent pathway (the inferior vestibular nerve), and the efferent pathway (the lateral vestibulospinal tract, the medial vestibulospinal tract, and the end muscle) [4]. Electronystagmography (ENG) is a gold standard vestibular function test. The caloric test induces vertigo and assesses only the horizontal semicircular canal function [5]. Compared to the ENG, VEMP testing is easier to perform, less complicated for interpretation, induces less dizziness or nausea, and is more tolerable to patients [6].

Stimuli that have been used to evoked VEMP responses include air and bone-conducted tone bursts, air-conducted clicks, forehead taps, and galvanic stimulation (short-duration transmastoid direct current stimulation) [4, 7]. Maintenance of tonic contraction of the SCM muscle during the test is a critical factor to elicit VEMP responses [8]. If the muscle is not contracted sufficiently, the VEMP responses may be absent.

Testing position to activate the SCM muscle included sitting with head turned, supine, recumbent, and prone positions with head lift or head turned [9–11]. Target EMG level to maintain tonicity of the muscle throughout the test with minimum patient discomfort is variable and depends on the test position [12, 13]. 

This study presents normative values of VEMP parameters using the authors' protocol.

The authors also reviewed methodology of VEMP recording and VEMP response parameters, which have been reported in the literature. The data should be beneficial to clinicians as a reference guide to set up VEMP testing and the interpretation of the VEMP responses in patients with vertigo or loss of balance.

## 2. Material and Methods

Fifty volunteers, whose age ranged from 18 to 60 years, with no history of hearing loss, vestibular or neurological disorders were recruited. All volunteers had a normal otoscopic examination and a normal pure tone audiometric threshold. Twelve men and thirty-eight women with age ranging from 22 to 57 years (44.0 ± 9.3; mean ± SD) enrolled in the study.

 After skin preparation, the active surface electrode was placed over the middle of the SCM, and the reference electrode was placed over the upper sternum. The ground electrode was placed at the forehead. Air-conducted alternating 500 Hz tone bursts (duration 5 msec) were presented unilaterally via an ER3A-inserted earphone (Etymotic Research, Elk Grove Village, IL, USA.) while the volunteer was sitting and turning his head to the contralateral side. A constant tonic activation of the SCM muscle was maintained at 30–75 *μ*V with visual feedback. The EMG signals were amplified (5000X), filtered (bandpass 10–1500 Hz with a Blackman gating function), and recorded (Intelligent Hearing System, Miami, Florida, USA). The stimulus intensity was started at 120 dBSPL (98 dBnHL). Response thresholds were determined using a down 10, up 5 dB step procedure. A minimum of two VEMP responses from 200 stimuli were averaged and calculated within −20 to 80 msec time window at 120 dBSPL.

The interaural amplitude difference (IAD) ratio was calculated by dividing the inter-ear difference of p1-n1 interamplitude by the sum of the p1-n1 interamplitude of both ears [13, 14]. The VEMP response threshold, p1 latency, n1 latency, p1-n1 interlatency, p1-n1 interamplitude, absolute inter-ear difference, and IAD ratio were analyzed using SPSS (SPSS Inc., Chicago, IL, USA). The protocol was approved by the Ethics Committee, Faculty of Medicine, Chiang Mai University. The study was conducted with the understanding and the consent of all subjects.

## 3. Results

The duration for testing in each subject ranged from 10 to 31 minutes (22.5 ± 5.1; mean ± SD). The VEMP responses presented in 86 of 100 ears in 50 volunteers, which elicited a response rate at 86%. Thirty-nine cases had bilateral VEMP response. Eight cases had unilateral VEMP responses. Three cases showed no VEMP responses in both ears. There was no difference between VEMP parameters of the right and the left ear.

**Figure 1:**
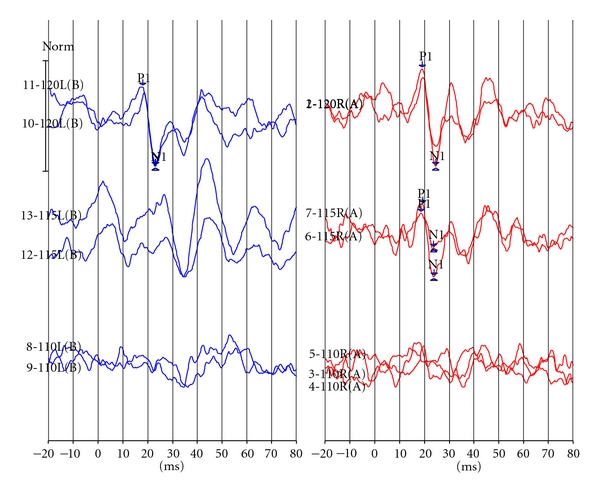
VEMP response waves of the left (threshold = 120 dBSPL) and the right ear (threshold = 115 dBSPL).

The VEMP response waves are shown in Figure 1. The VEMP parameters are shown in Tables 1 and 2.

## 4. Discussion

The recommended protocol of the VEMP testing in this study was using air-conducted alternating 500 Hz tone-bursts, starting at 120 dBSPL (98 dBnHL), presented monoaurally while the subject was sitting and turning head to the contralateral side. Variety of the protocols and normative values of VEMP responses from several studies are shown in Tables 3 and 4. The differences in protocols including stimulation type, stimulus intensity, number of stimuli, testing position, method of the SCM activation, electrode montage, and EMG level resulted in difference normative values of the VEMP testing

The air-conducted tone burst at 500 Hz, and clicks were the most widely used stimuli. Acoustically responsive fibers in the vestibular nerve showed to be the most responsive to frequencies between 500 and 1000 Hz, with little to no responsiveness to auditory stimuli above 3000 Hz [28]. 

Optimal stimulus frequencies for VEMP testing have been reported at 300–350 Hz [29], 500 Hz [30], and 700 Hz [11]. With click stimulation, the intensity that was required to evoke VEMP was higher than tone burst [9, 11, 31] about 95–100 dB above normal hearing level (140–145 dBSPL), which are relatively uncomfortable for subjects [7, 30]. The VEMP results evoked by clicks were more scattered than tone burst [30]. Tone-burst-evoked VEMP responses had lower stimulus thresholds, larger amplitude than click-evoked ones [11, 27]. Tone burst stimulation at 500 Hz tone was considered as an ideal stimulation [13, 30], with the stimulus intensity that ranged between 95–105 dBnHL or 115–130 dBSPL (Table 3). Although the tone burst stimulation at 95 dBnHL was the most commonly used, the authors found that 98 dBnHL stimulus improved rate of the responses and was comfortable to the subjects. 

 The midpoint of the SCM muscle is the optimal location for recording VEMP. Although VEMP responses recorded from the upper part of the SCM muscle showed the largest amplitude compared to the locations at the level of mandibular angle, the middle part of the muscle, and immediately above sternal and clavicular origins of the SCM muscle, the amplitude was not consistent [32]. In authors' experiences, placing the electrodes over the most prominent part or at the upper half of the SCM muscle was a less constant distance than placing at the midpoint of the muscle. The distance between the mastoid tip and head of clavicle can be easily measured and divided into half. An exact location of the electrode over the SCM muscle provided a more consistent response between the right and the left side and among the subjects. The authors suggest placing the active surface electrode over the middle of the SCM. 

Maintaining sufficient tonicity of the muscle throughout the test with minimum patient discomfort was critical in VEMP recording. No response was recorded when the SCM was not activated [12]. The SCM muscle activation by turning the head in sitting position was sufficient to generate the VEMP responses without early fatigability. Methods to activate the SCM muscle bilaterally included (1) supine or recumbent position and elevation of the head and (2) sitting and pushing the forehead against a load cell; unilaterally included (1) supine and turning the head and (2) sitting and turning the head (Table 3). 

Directly monitored tonic EMG levels for the SCM muscle activation were varied: 30–50 *μ*V [12], 40–150 *μ*V [18], and 50–200 *μ*V [13]. In the authors' experience, raising the head from supine position and setting the EMG level higher than this study's protocol tended to discomfort and fatigue the subjects. It was uncommon to see the responses after 200 stimulus or higher. The longer the SCM contraction, the higher the EMG level and higher the number of the stimuli; the subjects required a longer resting time between each stimulation to get muscle relaxation. The testing time was then increased. Another method to control the SCM muscle contraction was pushing their chin against the inflatable cuff of a blood pressure manometer. The cuff was inflated to a cuff pressure of 20 mmHg, and subjects were instructed to press until 40 or 45 mmHg was reached [16, 33]. Without EMG monitoring available, clinicians may consider this alternative method for maintaining the SCM muscle contraction.

A comparison among the studies in Tables 3 and 4 showed that p1 latency and n1 latency of the tone burst stimulation were longer, the thresholds were lower, and the amplitudes were higher than of the click stimuli. P1-n1 amplitude showed wide range of normative values and standard deviations compared to the latencies. 

The response rate in this study was 86%, which is lower than the previous reports. The stimulus intensity in this study is 120 dBSPL. The mean ± SD of the threshold was 100–115.1 ± 4.6 dBSPL. The stimulus possibly is not intense enough. The response rates at 97% with 123 dBSPL [16], at 100% with 125 dBSPL [15], and 130 dBSPL [17] tone burst stimuli were reported. However, response rates at 100% with 115 dBSPL [9] and 88% with 95 dBnHL tone burst stimulation [24] were found. The lowest response rate at 33% [16] was evoked by 90 dBnHL clicks, which is the softest stimulus level in Table 4. With 95 dBnHL click stimulation [22], the response rate in older subjects (90%) was lower than that in younger subjects (98%). The fact that the response rate with 100 dBnHL (95%) [11] was lower than that with 95 dBnHL (100%) [19], click stimulation is possibly affected by age (ranging between 25–85 years and 27–33 years). To determine a normative value of VEMP in the clinic, limitation of enrolled subject's age is recommended.

With tone burst stimulation in a sitting, head turned position, the threshold tend to increase (112 (±6), 114.16 (±6.45), 115.1 (±4.6) dBSPL) if the stimulus level decreased (at 130, 123, and 120 dBSPL stimulation) ([16, 17] and this study in orderly). To evoke a good response rate or threshold of VEMP, the intensity of stimulus should be set at least at 125 dBSPL tone burst or 95 dBnHL clicks with 200 stimuli. 

The amplitude (28.36 ± 11.65 *μ*V) and target EMG level to maintain tonicity of the muscle (30–75 *μ*V) in this study were lower than other studies. One study, however, reported the amplitude at 198.53 ± 64.64 *μ*V with target EMG level at 50 *μ*V [19]. The higher level of amplitude was observed in the head raised position with the higher target EMG level. To enhance wave amplitude, target EMG level should be set up at least 50 *μ*V with minimum of its range in head raised position. The longer the latency of p1, the longer of n1 latency observed. The shortest p1 latency was shown in one study reporting the highest amplitude [14]. If the amplitude of VEMP is stabilized, the latency of p1 and n1 should be less variable. 

VEMP parameters generally used for interpretation were the presence or absence of a VEMP response, VEMP threshold, latency of p1 and n1, and p1-n1 interamplitude. This study also reported other VEMP parameters including p1-n1 interlatency, interaural difference of p1 and n1 latency, and interaural amplitude difference (IAD) ratio. The most helpful parameter for the interpretation of the abnormality of the VEMP responses should be further studied. 

## 5. Conclusion

Protocols to evoke VEMP responses and its norm were different in each individual clinic. The response rate, threshold, and VEMP parameters were reviewed and summarized. The authors encourage using VEMP testing as a battery of vestibular function tests in balance disorder patients using previous reports for evidence-based guidance. The VEMP is not a replacement for the caloric or ENG test.

## Figures and Tables

**Table 1:** VEMP parameters in 86 ears.

VEMP parameters	Range	Mean ± SD
Threshold (dBSPL)	100–120	115.1 ± 4.6
p1 latency (msec)	12.60–20.10	15.99 ± 2.04
n1 latency (msec)	19.70–27.60	23.08 ± 1.50
p1-n1 interamplitude (*μ*V)	10.12–71.38	28.36 ± 11.65
p1-n1 interlatency (msec)	4.10–13.10	7.10 ± 1.95

**Table 2:** Interaural differences of VEMP parameters in 39 cases.

VEMP parameters	Range	Mean ± SD
Interaural difference of threshold	0–10	3.59 ± 3.62
Interaural difference of p1 latency	0.10–5.30	1.75 ± 1.41
Interaural difference of n1 latency	0–3.40	1.20 ± 0.83
Interaural difference of p1-n1 interlatency	0.20–4.60	1.62 ± 1.20
Interaural difference of p1-n1 interamplitude	0.49–33.78	7.98 ± 6.85
Asymmetrical ratio (%)	0.67–32.98	14.22 ± 9.42

**Table 3:** VEMP protocols.

Author	Country	Position	Active/positive/noninverting	Reference/inverting	Ground	Stimulus	Intensity	No. of stimuli	EMG level
Isaradisaikul 2012 (referred to this study)	Thailand	Sitting, head turned	Midpoint of the SCM	The sternal notch	The forehead	STBs of 500 Hz	98 dBnHL (120 dBSPL)	200	30–75 *μ*V
Kerdsiri 2010 [15]	Thailand	Recumbent, head raised and turned	The sternum	Upper 1/3 of the SCM muscle	The forehead	STBs of 500 Hz	105 dBnHL (125 dBSPL)	80–150	50–300 *μ*V
Janky 2009 [16]	USA	Sitting, head turned	The SCM	The manubrium of the sternum	The forehead	STBs of 500 Hz Rarefaction clicks	123 dBSPL (80 dBnHL) 119 dBSPL (90 dBnHL)	200	45 mmHg (cuff)
Maes 2009 [17]	Belgium	Sitting, head turned	Midpoint of the SCM	The sternoclavicular junction	The forehead	STBs of 500 Hz	95 dBnHL (130 dBSPL)	256	40 mmHg (cuff)
Isaradisaikul 2008 [18]	USA	Recumbent, head raised and turned	The sternum	Midpoint of the SCM	The forehead	STBs of 500 Hz	110 dBHL (115.5 dBSPL)	100	40–150 *μ*V
Wu 2007 [19]	Taiwan	Supine, head raised	Upper half of the SCM	The sternal notch	The forehead	STBs of 500 Hz/ Rarefaction clicks	95 dBnHL	200	50 *μ*V
Kelsch 2006 [20]	USA	Supine, head raised	Midpoint of the SCM	The ipsilateral upper sternum	The contralateral neck	alternating clicks	90 dBnHL	150	NA
Wang 2006 [21]	Taiwan	Supine, head raised then turned	Upper half of the SCM	Lateral end of the upper sternum	NA	STBs of 500 Hz	95 dBHL	200	50–200 *μ*V
Basta 2005 [9]	Germany	Head turned	Midpoint of the SCM	The sternum	The forehead	STBs of 500 Hz	115 dBSPL	NA	50–200 *μ*V
Su 2004 [22]	Taiwan	Supine, head raised	Upper half of the SCM	Lateral end of the upper sternum	NA	Rarefaction clicks	95 dBnHL	128	50–200 *μ*V
Wang 2004 [23]	Taiwan	Supine, head raised	Upper half of the SCM	Lateral end of the upper sternum	NA	STBs of 500 Hz	105 dBHL	200	50–200 *μ*V
Cheng 2003 [24]	Taiwan	Supine, head raised	Upper half of the SCM	Lateral end of the upper sternum	NA	STBs of 500 Hz/ Rarefaction clicks	95 dBnHL	128	50–200 *μ*V
Wang 2003 [25]	Taiwan	Supine, head raised	Upper half of the SCM	Lateral end of the upper sternum	NA	STBs of 500 Hz	95 dBnHL	200	50–200 *μ*V
Brantberg 2001 [26]	Sweden	Supine, head raised	The most Prominent part of the SCM	Midpoint of the clavicle	The uppermost part of the sternum	Rarefaction clicks	100 dBnHL	128	NA
Ochi 2001 [14]	Japan	Head turned	Upper half of the SCM	Upper edge of the sternum	The forehead	Rarefaction clicks	95 dB	50	NA
Welgampola 2001 [27]	Australia	Recumbent, head raised	Upper 1/3 of the SCM	Medial ends of the clavicles	The sternum	Rarefaction clicks	100 dBnHL	256	NA
Wu 1999 [10]	Taiwan	Supine, head raised	Upper half of the SCM	Lateral end of the sternum	NA	STBs of 500 Hz	95 dBnHL	200	NA

**Table 4:** Normative values of VEMP (mean ± SD) from literature.

Author	*N* (M : F)	Age range (years)	Response rate (%)	Threshold	P latency (msec)	*N* latency	Amplitude (*μ*V)	AR
Isaradisaikul 2012 (referred to this study)	50 (12 : 38)	22–57	86%	115.1 (±4.6) dBSPL	15.99 (±2.04)	23.08 (±1.50)	28.36 (±11.65)	14.22 (±9.42)
Kerdsiri 2010 [15]	40 (18 : 22)	21–57	100%	113 (±6) dBSPL	13.60 (±1.27)	19.90 (±1.87)	117.51 (±55.15)	NA
Janky 2009 [16]	46	20–76	97%^1^	114.16 (±6.45) dBSPL	16.24 (±2.42)	22.97 (±2.62)	27.65 (±11.13)	NA
			33%^2^	122.17 (±4.09) dBSPL	13.62 (±2.88)	20.00 (±2.66)	27.17 (±9.13)	NA
Maes 2009 [17]	61 (28 : 23)	19–39	100%	112 (±6) dBSPL	14.97 (±1.42)	23.41 (±1.66)	147.34 (±68.66)	0.12 ± 0.10
Isaradisaikul 2008 [18]	20 (6 : 14)	24–49	87%	110.1 (±5.2) dBSPL	14.44 (±1.92)	21.16 (±2.11)	160.71 (±101.11)	18.8 (±16.5)
Wu 2007 [19]	22 (11 : 11)	17–30	100%^1^	NA	14.83 (±0.81)	22.54 (±1.30)	198.53 (±64.64)	0.13 (±0.12)
			100%^2^	NA	12.43 (±1.01)	19.85 (±1.65)	81.23 (±32.56)	0.20 (±0.13)
Kelsch 2006 [20]	30 (16 : 14)	3–11	93%	NA	11.3 (±1.3)	17.6 (±1.4)	122 (±68)	17.6 (±12.8)
Wang 2006 [21]	20 (14 : 6)	23–30	100%	78 (±7) dB	13.1 (±0.7)	20.3 (±1.3)	130.5 (70.8–262.0)	NA
Basta 2005 [9]	64 (26 : 38)	20–76	100%	NA	16.1 (±2.1)	23.8 (±2.2)	67.1 (±40.2) (20–40 yr)	NA
Su 2004 [22]	80 (46 : 34)	21–40	98%	NA	11.47 (±0.86)	19.05 (±1.31)	NA	0.19 (±0.15)
		41–60	90%	NA	11.59 (±0.79)	18.98 (±1.07)	NA	0.13 (±0.12)
Wang 2004 [23]	13 (10 : 3)	22–35	100%	88 (±10) dB	14.08 (±1.27)	20.66 (±1.52)	142.6 (81.5–239.0)	NA
Cheng 2003 [24]	29 (24 : 5)	17–43	88%^1^	NA	12.49 (±0.94)	19.79 (±1.40)	102.84 (±44.56)	NA
			98%^2^	NA	11.45 (±0.87)	19.17 (±1.55)	119.55 (±44.03)	NA
Wang 2003 [25]	14 (11 : 3)	24–32	100%	NA	14.49 (±1.28)	21.83 (±1.65)	NA	0.03
Brantberg 2001 [26]	23 (12 : 11)	22–42	NA	NA	11.40 (10.62–11.59)	18.18 (17.34–19.20)	66.6 (38.3–108.2)	NA
Ochi 2001 [14]	18 (9 : 9)	21–38	100%	95 dB	10.75 (±1.34)	19.92 (±2.43)	203.96 (±118.68)	12.6 ± 8.1
Welgampola 2001 [27]	70 (34 : 36)	25–85	95%	89.6 (±6.9) dBnHL	12.0 (±1.0)	20.3 (±1.7)	72.5 (±46.8)	21.60%
Wu 1999 [10]	16 (16 : 0)	27–33	100%	NA	16.6 (±1.5)	25.2 (±2.0)	54.6 (±28.9)	NA

Note: ^1^STBs of 500 Hz, ^2^rarefaction clicks.
